# Acute unilateral hearing loss as an unusual presentation of cholesteatoma

**DOI:** 10.1186/1472-6815-5-9

**Published:** 2005-09-18

**Authors:** Daniel Thio, Shahzada K Ahmed, Richard C Bickerton

**Affiliations:** 1 Department of Otorhinolaryngology, South Warwickshire General Hospitals NHS Trust Warwick CV34 5BW UK; 2 Department of Otorhinolaryngology, South Warwickshire General Hospitals NHS Trust Warwick CV34 5BW UK; 3 Department of Otorhinolaryngology, South Warwickshire General Hospitals NHS Trust Warwick CV34 5BW UK

## Abstract

**Background:**

Cholesteatomas are epithelial cysts that contain desquamated keratin. Patients commonly present with progressive hearing loss and a chronically discharging ear. We report an unusual presentation of the disease with an acute hearing loss suffered immediately after prolonged use of a pneumatic drill.

**Case presentation:**

A 41 year old man with no previous history of ear problems presented with a sudden loss of hearing in his right ear immediately following the prolonged use of a pneumatic drill on concrete.

The cause was found to be a fractured long process of incus which had been eroded by the presence of an attic cholesteatoma.

A tympanomastoidectomy and ossiculoplasty was performed with good result.

**Conclusion:**

Cholesteatomas may be asymptomatic and insidious in their onset. This case illustrates the point that an indolent disease such as this may present in unusual ways and the clinician must always have a high index of suspicion combined with thorough assessment and examination of every patient.

## Background

The definition of cholesteatoma is the occurrence of keratinizing, stratified, squamous epithelium within the middle-ear cavity where otherwise only modified respiratory epithelium ought to be present [1]. Patients normally present with a chronically discharging ear and may complain of hearing loss. We present the case of a 41 year old man whose primary presentation of cholesteatoma was a sudden unilateral hearing loss following extended use of a pneumatic drill – a feature not associated with chronic middle ear disease. This mode of presentation has never previously been reported in the literature.

## Case presentation

A 41-year-old gentleman was referred to the otolaryngology outpatient clinic with a one-month history of acute right-sided hearing loss. At the time of onset, he noticed a sudden loss of hearing in his right ear immediately following the prolonged use of a pneumatic drill on concrete without the benefit of ear protection. There was no previous history of noise exposure or any ear problems.

**Figure 1:**
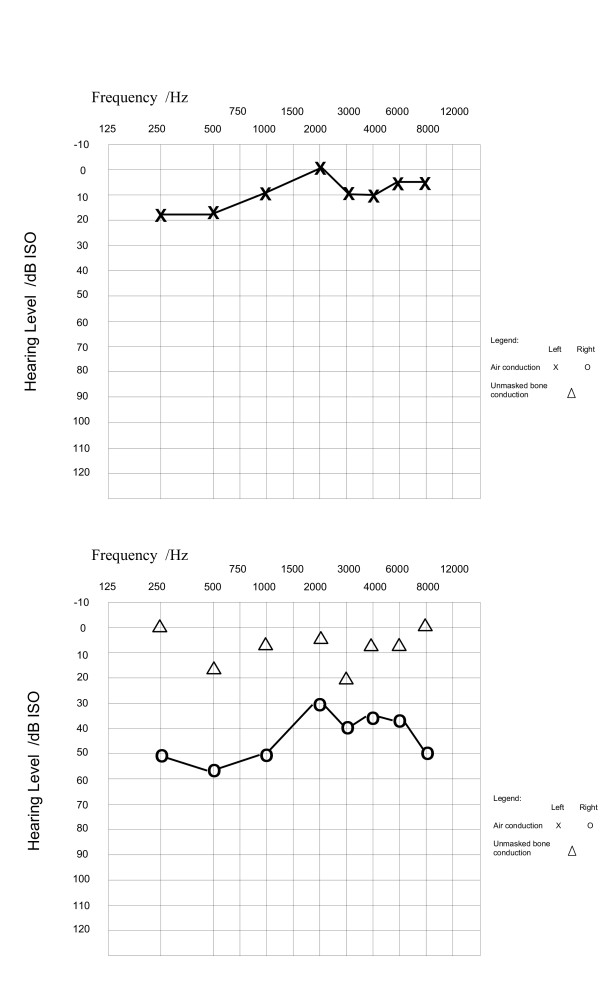
Pre-operative pure tone audiogram.

Assessment of the right tympanic membrane revealed a retraction pocket in the attic containing dry cholesteatoma with the suspicion of the cholesteatoma passing postero-inferiorly. His left tympanic membrane was normal at otoscopy. Weber testing lateralised to the right side and Rinne's test was negative on the right using a 512Hz tuning fork. The rest of the examination was unremarkable. Pure tone audiometry confirmed a 45-decibel mean conductive hearing loss on the right (Figure 1) with normal hearing levels in the left ear.

**Figure 2:**
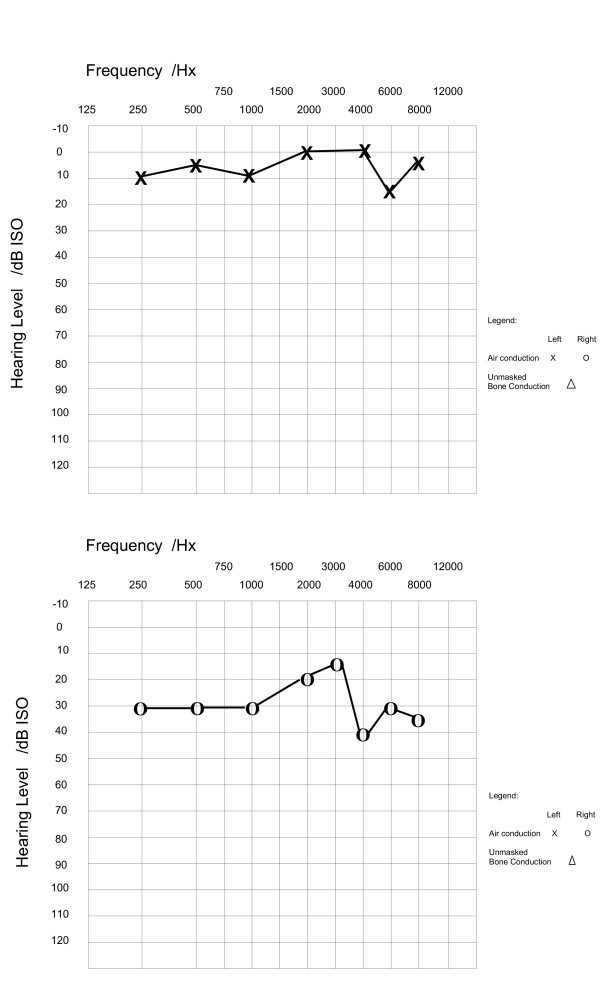
Post-operative pure tone audiogram.

He subsequently underwent a right tympanomastoidectomy and ossiculoplasty. Operative findings confirmed extensive erosion and fracture of the long process of incus (LPI) by a moderate size cholesteatoma. The stapes footplate was mobile. Following careful dissection and removal of the entire cholesteatoma together with the head of the malleus, ossicular continuity was restored with a Goldenberg partial ossicular replacement prosthesis connecting the stapes suprastructure to the handle of malleus. A repeat audiogram 7 weeks post-surgery showed a 15-decibel improvement in his right ear (Figure 2). The patient went on to make a good recovery with no recurrence of cholesteatoma at 12 months with good hearing thresholds

## Discussion

The history of acute noise exposure without ear protection may normally be expected to result in a bilateral sensorineural hearing loss. Furthermore, our patient had no previous history of otological problems, which is unusual in somebody with such extensive cholesteatoma. His presenting complaint of sudden hearing loss was a result of vibration from the pneumatic drill, fracturing the already eroded long process of incus (LPI), thus resulting in immediate ossicular discontinuity and the subsequent sudden hearing loss. This was confirmed at operation.

Cholesteatomas are keratin-containing epidermoid cysts that classically arise from the pars flaccida or postero-superior segment of the tympanic membrane. They have the propensity to expand into the middle ear cleft (MEC) and beyond, leading to both intracranial and extracranial complications. This is commonly compounded by the presence of infection.

Clinical features vary and arise as a result of the disease itself or its complications. The most common presenting features are an offensive smelling discharge and hearing loss [2]. Hearing loss may be a feature due to ossicular chain disruption or may result from accumulation of toxic inflammatory mediators which pass through the round window into the cochlea. Dizziness and vertigo may be due to the presence of a labyrinthine fistula [3].

Signs include crusting in the attic region, granulomatous polyps and marginal granulomas, the presence of keratin debris in a retraction pocket, and marginal and attic perforations [3,4].

Management of cholesteatomas is primarily surgical. The aim of surgery is to convert unsafe disease to safe disease, with restoration of hearing a secondary priority. Surgery is directed toward the eradication of entrapped keratinising epithelium and debris from the middle ear and mastoid air spaces and tympanomastoidectomy is the operation of choice [5]. The decision as to whether to perform an ossiculoplasty at the initial operation depends on the findings at surgery and the surgeon's preference.

## Conclusion

This case emphasises the point that cholesteatomas can be largely asymptomatic. It was fortuitous that vibrations from a pneumatic drill caused the already eroded LPI to fracture, facilitating the diagnosis and treatment of this indolent and potentially dangerous condition. A presentation of such unlikely coincidences has not been reported in the literature. The clinician must always have a high index of suspicion combined with thorough assessment and examination of every patient to ensure that this indolent disease is not missed.

## Competing interests

The author(s) declare that they have no competing interests.

## Authors' contributions

DT drafted the manuscript and prepared the figures

SKA revised the manuscript

RCB performed the ossiculoplasty and proof-read the manuscript

All authors have read and approved of the manuscript

## Pre-publication history

The pre-publication history for this paper can be accessed here:
